# Identification and verification of potential biomarkers in sertoli cell-only syndrome via bioinformatics analysis

**DOI:** 10.1038/s41598-023-38947-4

**Published:** 2023-07-27

**Authors:** Yuting Jiang, Xiao Yang, Linlin Li, Xin Lv, Ruixue Wang, Hongguo Zhang, Ruizhi Liu

**Affiliations:** Reproductive Medicine Center, Prenatal Diagnosis Center, First Hospital of Jilin University, No. 1 Xinmin Street, Changchun, 130021 China

**Keywords:** Computational biology and bioinformatics, Molecular biology, Molecular medicine

## Abstract

Sertoli cell-only syndrome (SCOS), a severe testicular spermatogenic failure, is characterized by total absence of male germ cells. To better expand the understanding of the potential molecular mechanisms of SCOS, we used microarray datasets from the Gene Expression Omnibus (GEO) and ArrayExpress databases to determine the differentially expressed genes (DEGs). In addition, functional enrichment analysis including the Gene Ontology (GO) and Kyoto Encyclopedia of Genes and Genomes (KEGG) was performed. Protein–protein interaction (PPI) networks, modules, and miRNA-mRNA regulatory networks were constructed and analyzed and the validation of hub genes was performed. A total of 601 shared DEGs were identified, including 416 down-regulated and 185 up-regulated genes. The findings of the enrichment analysis indicated that the shared DEGs were mostly enriched in sexual reproduction, reproductive process, male gamete generation, immune response, and immunity-related pathways. In addition, six hub genes (CCNA2, CCNB2, TOP2A, CDC20, BUB1, and BUB1B) were selected from the PPI network by using the cytoHubba and MCODE plug-ins. The expression levels of the hub genes were significantly decreased in patients with SCOS compared to that in normal spermatogenesis controls as indicated by the microarray data, single-cell transcriptomic data, and clinical sample levels. Furthermore, the potential miRNAs were predicted via the miRNA-mRNA network construction. These hub genes and miRNAs can be used as potential biomarkers that may be related to SCOS. However, it has not been proven that the differential expression of these biomarkers is the molecular pathogenesis mechanisms of SCOS. Our findings suggest that these biomarkers can be serve as clinical tool for diagnosis targets and may have some impact on the spermatogenesis of SCOS from a testicular germ cell perspective.

## Introduction

Infertility affects 10–15% of reproductive-age couples worldwide, and males are responsible for approximately 50% of these cases^1^. Non-obstructive azoospermia (NOA) is the most severe form of male infertility and testicular spermatogenic failure is the most typical phenotype of NOA. Chromosomal abnormalities (e.g. Klinefelter syndrome) or Y chromosome microdeletions are responsible for the genetic etiology of 20–30% of NOA patients. However, approximately 70% of the etiology of these patients remains unknown to date^2,3^. NOA is classified into three categories based on the histopathological evaluation of the testicular biopsy: hypospermatogenesis (HS), maturation arrest (MA), and SCOS^4^.

SCOS is characterized by the complete absence of male germ cells (GCs) and is probably caused by the disruption of male GCs development in the fetal and/or early postnatal stages. Spermatogenesis is a complex process of spermatozoa generation, which involves meiosis and spermiogenesis. Through spermatogenesis, diploid cells to differentiate into mature and motile (haploid) spermatozoa^5^. Accumulating evidence reveals that in addition to providing the blood-testis barrier (BTB) and nurturing the microenvironment for spermatogenesis, the Sertoli cells (SCs) play the central role in testicular spermatogenesis^6,7^. However, immature SCs undergo continuous proliferation and compromised differentiation, resulting in increased germ cell apoptosis and infertility. This may result in initiation and progression of SCOS^8^. In the rare cases of SCOS, a few spermatozoa can be detected by microsurgical Testicular Sperm Extraction (micro-TESE) enabling for the further implementation of intracytoplasmic sperm injection (ICSI). However, for all the patients in whom sperm retrieval is not successful, Artificial Insemination by Donor (AID) remains the only option^9^. Hence, further investigation is warranted regarding the underlying pathogenesis of SCOS, encompassing etiology, progression, distinctive presentations and prospective biomarkers. Investigating the testicular DEGs associated with SCOS can serve to elucidate and enhance our overall comprehension of the SCOS pathology.

Currently, with the rapid development of transcriptional profiling technologies, more than 1.5 million samples have been included in databases such as the NCBI Gene Expression Omnibus (GEO) and EBI ArrayExpress^10^. Furthermore, bioinformatic analysis can be performed to determine the pivotal genes and signaling pathways of diseases to develop therapeutic approaches. However, the public databases of SCOS and normal control are limited. Almost all research data needs to be extracted from NOA-relevant datasets, which leads to inconsistent results owing to the small sample size. In addition, most of these studies lack independent validation. Thus, the identification and validation of the hub genes for SCOS is particularly important. In the current study, the mRNA expression data of testicular samples from the GEO and ArrayExpress databases were analyzed to determine the potential DEGs between SCOS and control groups. Moreover, functional enrichment analysis was performed in addition to constructing protein–protein interaction (PPI) and miRNA-mRNA networks. Thereafter, the identified hub genes were subjected to RT-qPCR analysis for further validation in clinical patients with SCOS. This study may make a contribute to comprehending the potential molecular mechanisms of SCOS through the utilization of bioinformatic analysis.

## Materials and methods

### Microarray data collection

**Figure 1:**
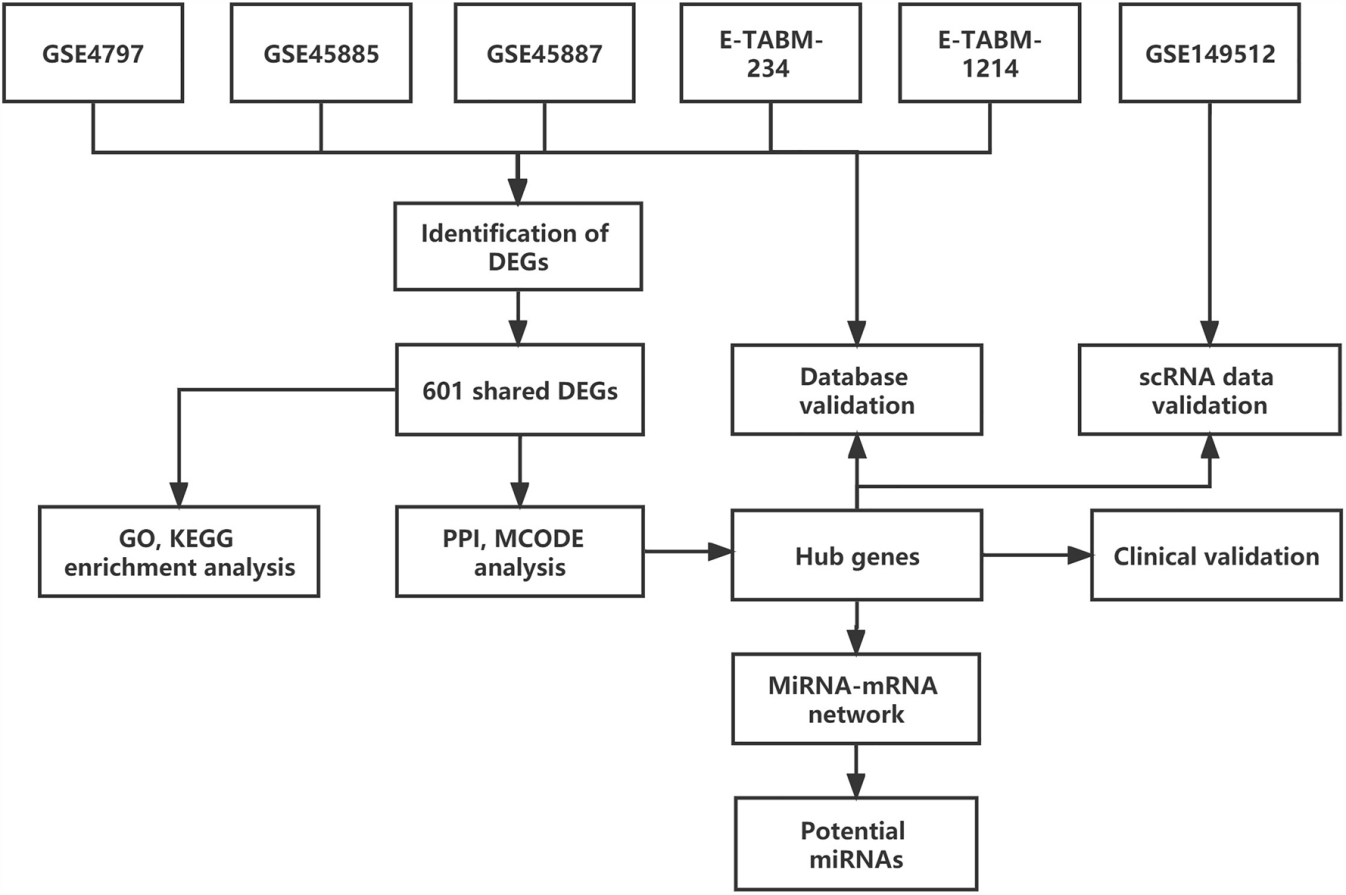
Flowchart of the study design. DEGs, differentially expressed genes; GO, gene ontology; KEGG, Kyoto Encyclopedia of Genes and Genomes; MCODE, Minimal Common Oncology Data Elements; PPI, protein–protein interaction; SCOS, Sertoli cell-only syndrome.

The complete microarray data were download from the GEO (https://www.ncbi.nlm.nih.gov/geo/) database and ArrayExpress (http://www.ebi.ac.uk/arrayexpress/) databases. According to the clinical characteristics and diagnostic criteria of the patients, the datasets that matched the following criteria were included in the study: (1) the gene sets must include SCOS and control groups; (2) the related patients with SCOS and normal controls underwent the testicular histopathological examination by Johnsen Score (SCOS, JS = 2; nomal control, JS = 10)^11^; and (3) raw data or series matrix file is available in the datasets. Finally, five microarray datasets were included for subsequent analysis: GSE4797, GSE45885, GSE45887, E-TABM-234, and E-TABM-1214. These datasets included 32 patients with SCOS and 35 control patients. The information on the accession number, microarray platform, and the selected samples of datasets are shown in Table 1. The annotation files for platforms were also downloaded from the GEO and ArrayExpress databases. The flowchart of the study is shown in Fig. 1.

**Table 1:** Characteristics for selected microarray datasets.

Accession	Platforms	Total samples	Selected SCOS samples
GSE4797	GPL2891	17 NOA vs. 9 control	5
GSE45885	GPL6244	27 NOA vs. 4 control	7
GSE45887	GPL6244	16 NOA vs. 4 control	5
E-TABM-234	GPL570	30 NOA vs. 8 control	8
E-TABM-1214	GPL570	30 NOA vs. 9 cryptorchidism vs. 8 control	7

### Differential expression analysis

Raw microarray data from the selected datasets were also processed and normalized with the “Bioconductor package” in the statistical software R. The limma package in R^12^ was used to identify the DEGs between the SCOS group and control group. The DEGs were identified based on the criteria of |log_2_ (FC)| ≥ 1 and adjusted p-value < 0.05. Thereafter, volcano plots were generated with the “volcanoplot” function in the Limma package.

### Functional enrichment analysis of shared DEGs

The Gene Ontology (GO)^13^ and Kyoto Encyclopedia of Genes and Genomes (KEGG)^14^ enrichment analysis were performed by the clusterProfiler R package (version 3.14.3)^15^. The minimum and maximum gene sets were set to 5 and 5000, respectively, and the *P*-value of < 0.05 was considered statistically significant. The GO terms, which list genes, can be clustered based on biological process (BP), molecular function (MF,) and cellular component (CC)^16^ and were visualized by the “ggplot2” R packages. KEGG pathways were visualized by the “GOplot2” R packages^17^.

### PPI network analysis and hub gene identification

The PPI network of all shared DEGs was constructed through the online STRING database (http://www.string-db.org/), and the confidence interaction score was set as 0.9 (highest confidence) in the PPI analysis. The network was imported into the Cytoscape software (v3.9.0) to construct the PPI network for better visualization and further identification of hub genes. The Molecular Complex Detection (MCODE)^18^ plug-in in Cytoscape used to illuminate the most interacting modules in the aforementioned PPI network above. The criteria were set as follows: max depth = 100; k-core = 4; node score cutoff = 0.2; degree cutoff = 4. The gene clusters with scores > 5 were focused on. The hub genes in the network were identified by cytoHubba^19^. Four algorithms (MCC, EPC, MNC, and Degree) were used to identify the top 10 core genes. Finally, the results of the four algorithms were crossed to obtain the final hub genes.

### Database validation of hub genes

The hub genes were validated by using the data from the aforementioned microarray datasets (GSE4797, GSE45885, GSE45887, E-TABM-234, and E-TABM-1214). Further validation of the hub genes was performed using single-cell transcriptomic data from GSE149512. The validation analysis involved scRNA-Seq datasets from three patients with SCOS and four normal adults from GSE149512. Cell type identification and clustering analysis were performed using the R package “Seurat” (v.3.1.2)^20^. Data preprocessing in this study involved calculating the percentage of gene numbers, cell counts, and mitochondrial sequencing counts. Genes with less than three cells detected were excluded, and genes with fewer than 200 cells detected were disregarded. To reduce dimensionality, the data were normalized and the “JackStraw” function was used to perform the principal component analysis (PCA). Applying a shared nearest neighbor modularity-optimization algorithm, the principal clusters were visualized using uniform manifold approximation and projection (UMAP). To identify the cell types, the marker genes from the clusters were matched with the CellMarker database (http://xteam.xbio.top/CellMarker/)^21^. The next step was to set the parameter resolution to 0.6 for the “FindClusters” function to evaluate the clustering of cells in normal adults and SCOS patient testes into different cell clusters, which included germ cells and somatic cells^22^.

### Clinical samples

A total of 10 patients with SCOS (JS = 2) and nine OA control patients (JS = 10) from the Reproductive Medicine Center, Prenatal Diagnosis Center, First Hospital of Jilin University (Changchun, China) were included in the study. All the patients underwent micro-TESE and the histopathological evaluation of the testicular biopsy was performed. Other testicular biopsy samples were flash-frozen in liquid nitrogen and stored at − 80 °C until RNA extraction was performed. Chromosomal karyotype abnormalities and Y chromosomal microdeletions were excluded. The clinical characteristics of the validation patients are shown in Supplementary Table 1.

### Reverse transcription-quantitative polymerase chain reaction (RT-qPCR)

Briefly, total RNA was extracted from human testis samples using TRIzol (CWBIO, Beijing, China). Reverse transcription expression was performed using RecertAid MM (Thermoscientific, Vilnius, Lithuania). The RT-qPCR was performed using the SYBR Green Master Mix (Thermoscientific, California, USA) following the manufacturer’s instructions. β-actin was used as an internal control. The relative expression level of genes was analyzed by the 2^−ΔΔCt^ method from three independent experimental repeats^23^. The primer sequences of six hub genes and β-actin are shown in Table 2.

**Table 2:** Primer sequences of hub genes for qRT-PCR.

Gene	Primer sequences
CCNA2	F: CACTCTACACAGTCACGGGA
	R: AGTGTCTCTGGTGGGTTGAG
CCNB2	F: TTACTGCTCTGCTCTTGGCTTC
	R: TCTCGGATTTGGGAACTGGTATAAG
TOP2A	F: CATTGAAGACGCTTCGTTATGG
	R: CAGAAGAGAGGGCCAGTTGTG
CDC20	F: TCGCATCTGGAATGTGTGCT
	R: CCCGGGATGTGTGACCTTTG
BUB1	F: AGCCCAGACAGTAACAGACTC
	R: GTTGGCAACCTTATGTGTTTCAC
BUB1B	F: AAATGACCCTCTGGATGTTTGG
	R: GCATAAACGCCCTAATTTAAGCC
β-actin	F: GGAGATTACTGCCCTGGCTCCTA
	R: GACTCATCGTACTCCTGCTTGCTG

### MiRNA-mRNA networks construction

To explore the potential interactions of miRNA and target genes, the miRNA–mRNA networks were predicted using the miRNet database 2.0 (https://www.mirnet.ca/). The miRNA target gene data were collected from four well-annotated databases: miRTarBase v8.0^24^, TarBase v8.0^25^ and miRecords^26^. The miRNA set libraries (Function, Disease, Transcription Factor, Cluster) were collected from TAM 2.0^27^. A *P*-value < 0.01 was used to screen for significant GO terms. The criteria of the miRNAs were set as only expressed in the human testicular tissues. The networks were visualized using Cytoscape.

### Ethical approval

In this study, the of RT-qPCR validation using by clinical samples was approved by the Ethics Committee of the First Hospital of Jilin University (Ethical Committee Reference NO. 2021-741). In addition, all patients read and signed the informed consent form. Other data in this study were downloaded from GEO and ArrayExpress databases for secondary data analysis. The current study conformed to all the guidelines and principles stated in the Declaration of Helsinki.

### Statistical analysis

The quantitative data were presented as the Mean ± SD. The non-parametric test was used to analyze the differences between the two groups. A *P*-value < 0.05 was considered statistically significant. All analyses were performed using R software (version 4.1.0).

## Results

### Identification of DEGs

**Figure 2:**
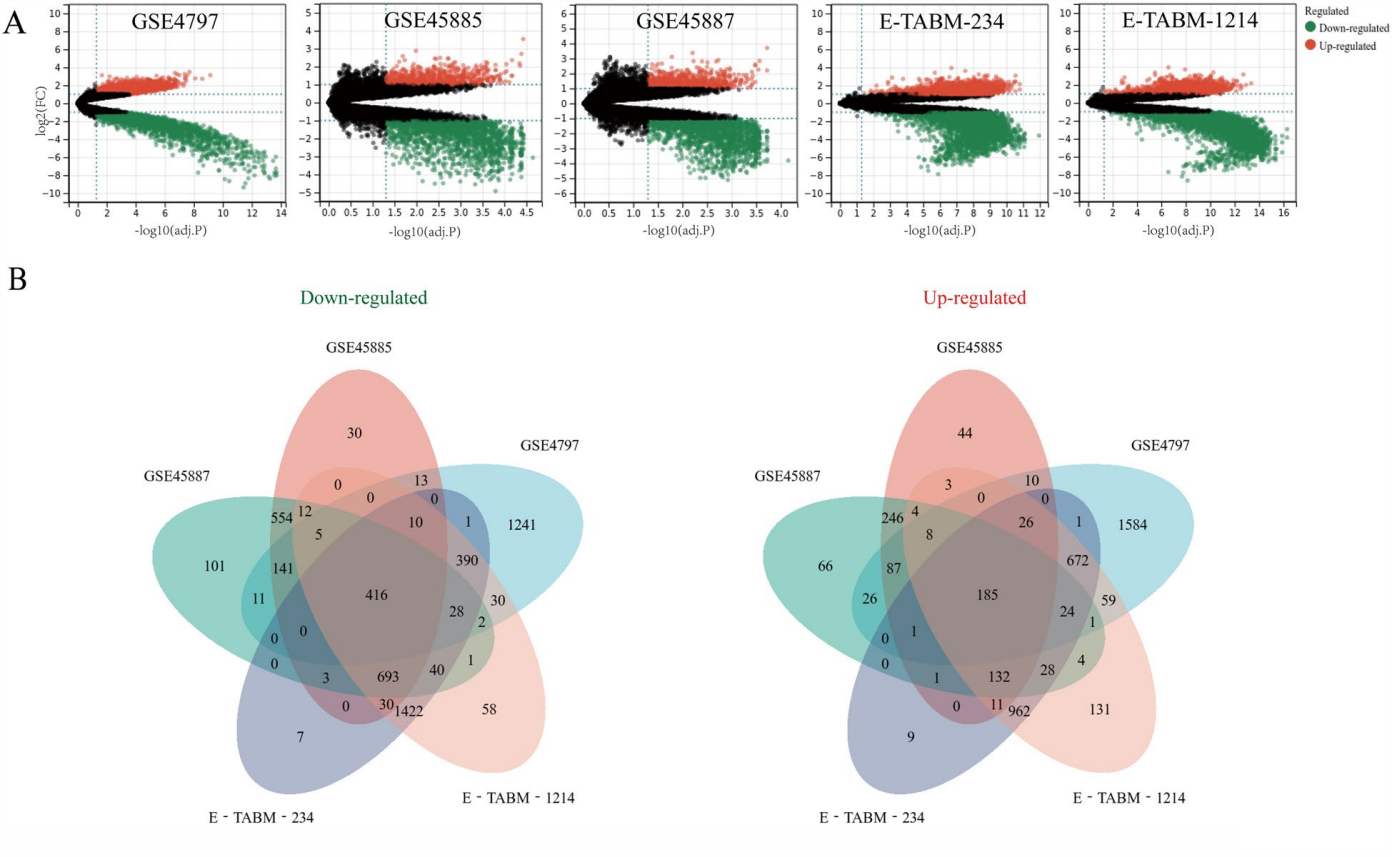
Results of DEGs analysis. (**A**) Volcano plots showing DEGs of the five microarrays. Red points represented significantly up-regulated genes, while green points represented significantly down-regulated genes, grey points represented genes without significant difference. The criteria of DEG is |logFC| > 1 and adj. *P*-value < 0.05. (**B**) Venn diagram depicting 416 down-regulated and 185 up-regulated overlapping genes among the five microarrays datasets.

The five microarray expression datasets were downloaded from the two aforementioned databases and the DEGs between SCOS and the normal control group were analyzed. The raw data successively underwent processes including quality control, background correction, and normalization. Based on the criteria adj. p < 0.05 and |logFC| ≥ 1, 2864, 758, 813, 2052, and 2250 genes were found to be significantly up-regulated in GSE4797, GSE45885, GSE45887, E-TABM-234, E-TABM-1214, respectively. Furthermore, 2228, 1907, 2007, 3040, and 3137 genes were significantly down-regulated in GSE4797, GSE45885, GSE45887, E-TABM-234, and E-TABM-1214, respectively. The DEGs of each comparison group are shown as volcano plots in Fig. 2A. These DEGs present in all five datasets were identified as consistent DEGs in SCOS. Finally, 601 shared DEGs (416 down-regulated and 185 up-regulated) were found and are presented in the Venn diagram (Fig. 2B). DEGs in each microarray dataset are shown in Supplementary Table 2.

### Functional and pathway enrichment of DEGs

**Figure 3:**
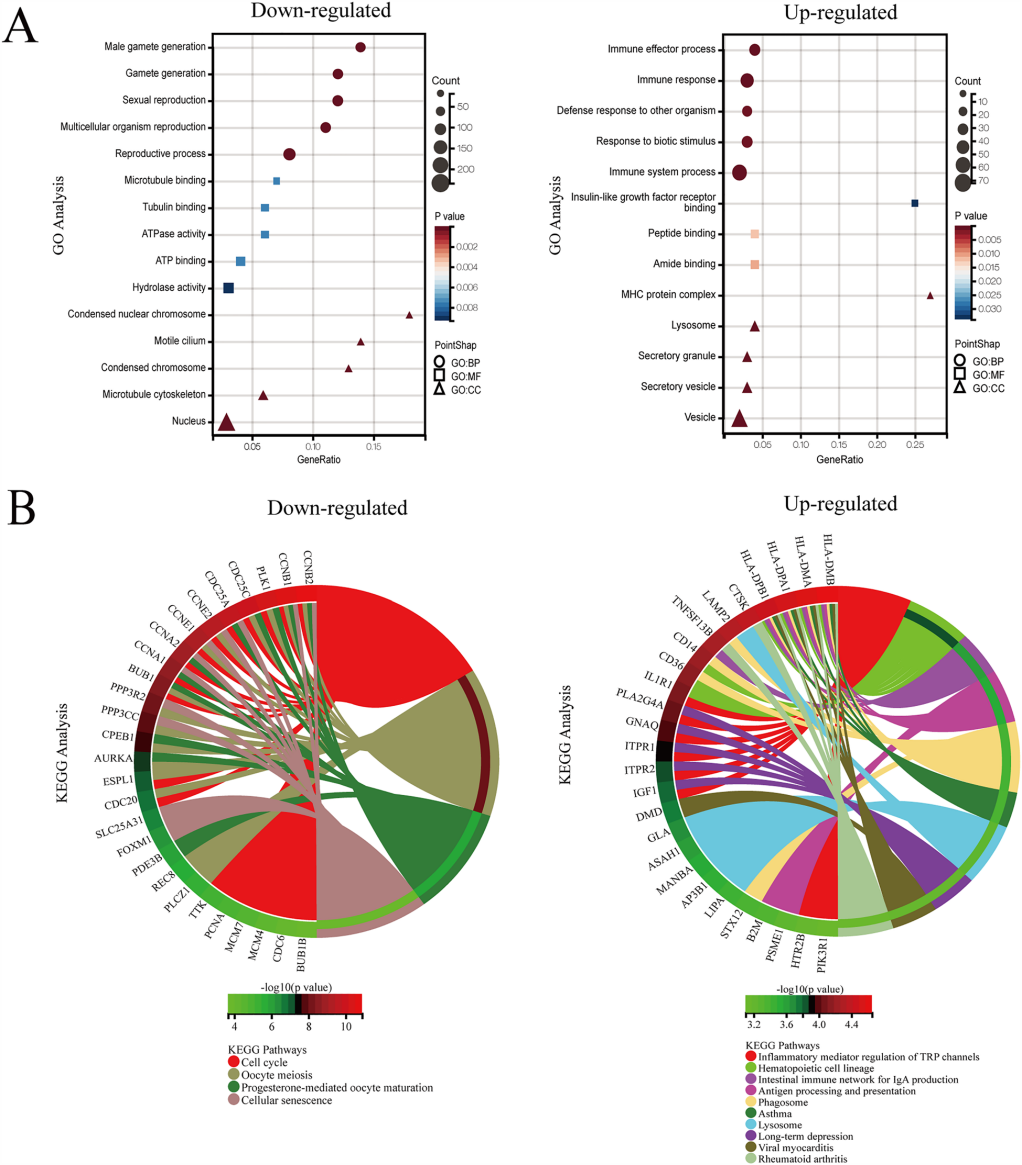
GO and KEGG enrichment of DEGs with the cut off criteria of *P*-value < 0.05. (**A**) Down-regulated (left) and up-regulated (right) DEGs in three parts of GO enrichment (BP, CC, and MF with different shape). The bubble color represents *P*-value in enriched pathways, and the size of the bubble represents the gene number. (**B**) Down-regulated (left) and up-regulated (right) DEGs of KEGG pathways enrichment. GOplot of the relationship between the list of selected genes and their corresponding KEGG pathways, together with the logFC of the genes. DEG, differentially expressed gene; GO, Gene Ontology; BP, biological process; CC, cellular component; MF, molecular function.

To explore the functions of DEGs, the 601 shared DEGs were uploaded for the GO and KEGG enrichment analysis. As the left-hand image in Fig. 3A shows, sexual reproduction, reproductive process, male gamete generation, gamete generation, multicellular organism reproduction, ATP binding, microtubule binding, tubulin binding, ATPase activity, hydrolase activity, nucleus, motile cilium, microtubule cytoskeleton, condensed chromosome, and condensed nuclear chromosome were the top five enriched GO terms in the BP, MF, and CC of down-regulated DEGs, respectively. In the up-regulated genes (Fig. 3A, right), immune response, immune system process, immune effector process, defense response to other organisms, response to biotic stimulus, lysosome, vesicle, secretory vesicle, secretory granule, MHC protein complex were the top five enriched GO terms in BP and CC, respectively. Only three enriched GO terms appeared in the MF of up-regulated DEGs: amide binding, peptide binding, and insulin-like growth factor receptor binding. Considering the KEGG pathway, four pathways were found to be enriched in the down-regulated DEGs, and 37 pathways were found to be enriched in the up-regulated DEGs, with the criteria of *P*-value < 0.05. The top 10 enriched pathways of up-regulated genes and four completely enriched pathways of down-regulated genes are shown in Fig. 3B. The genes shared in the pathways are listed on the left side of the circular plates. The results of GO and KEGG pathway enrichment analysis are shown in Supplementary Table 3.

### Identification of gene clusters and hub genes

**Figure 4:**
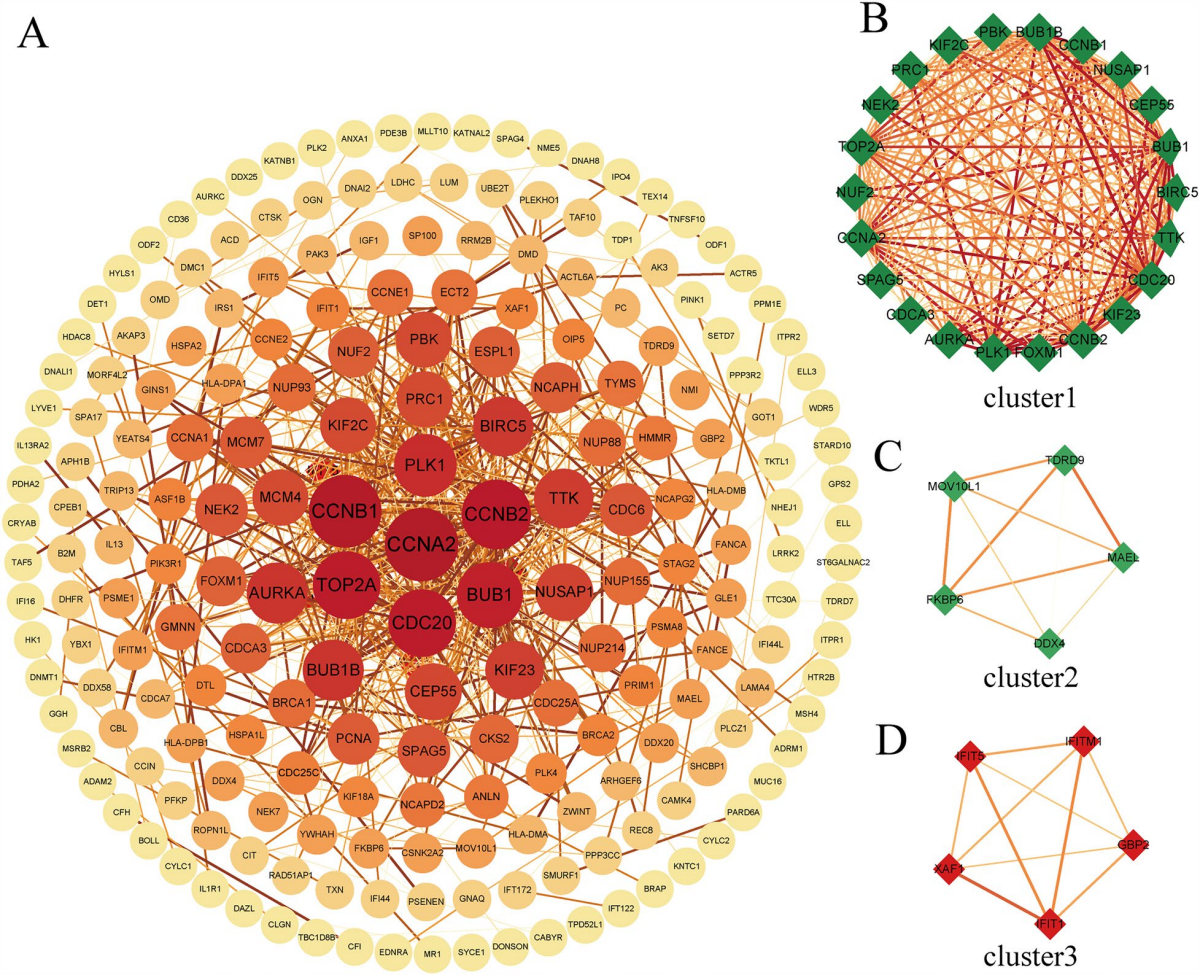
The PPI network analyzed by cytoHubba and MCODE. (**A**) PPI network encoded by hared DEGs, consisting of 211 nodes and 642 edges. The value of the gene degree is represented by the size of the node. (**B**–**D**) Three cluster modules extracted by MCODE. PPI, protein–protein interaction; MCODE, Minimal Common Oncology Data Elements.

With confidence > 0.9 (highest confidence), 601 shared DEGs were uploaded to the STRING website. A PPI network containing 211 nodes and 642 edges was constructed and visualized by Cytoscape as shown in Fig. 4A. The size and color of the nodes indicated the level of neighborhood connectivity, and the magnitude of the nodes indicated the value of a degree. The color of each edge between the two proteins denotes the confidence level from STRING analysis. The interactions of the proteins are shown in Supplementary Table 4. The “MCODE” plug-in was used to explore the highly connected modules, and clusters with scores ≥ 5 were focused on. Three functional subnet modules were selected from the PPI network. The top-scoring MCODE cluster containing 22 nodes and 210 edges (MCODE score of 20) is shown encircled within the whole STRING network in cluster 1 (Fig. 4B). Five nodes and 10 edges were present in cluster 2 (Fig. 4C) and cluster 3 (Fig. 4D) respectively, where the red node indicates up-regulated, while the green node indicates down-regulated gene. The nodes, edges and scores of the three modules from MCODE are shown in Supplementary Table 5.

CytoHubba in Cytoscape was used to determine hub genes and identify them by crossing the results of the four algorithms, including MCC, EPC, MNC, and Degree. The results of the CytoHubba are shown in Supplementary Table 6. After calculation, the one that intersected the four algorithms was considered, and finally, six down-regulated genes that appeared among the top ten genes were regarded as the most likely hub genes. The six hub genes are as follows: CCNA2, CCNB2, TOP2A, CDC20, BUB1, and BUB1B. The details of the hub genes are shown in Table 3.

**Table 3:** Hub genes screened by four algorithms of cytoHubba.

Gene symbol	Description	log2FC				
		GSE4797	GSE45885	GSE45887	E-TABM-234	E-TABM-1214
CCNA2	Cyclin A2	− 3.776	− 1.100	− 1.398	− 2.662	− 2.939
CCNB2	Cyclin B2	− 6.560	− 3.196	− 3.896	− 5.300	− 5.816
TOP2A	DNA topoisomerase II alpha	− 2.854	− 2.270	− 2.930	− 5.135	− 5.732
CDC20	Cell division cycle 20	− 5.276	− 2.796	− 3.177	− 4.712	− 5.173
BUB1	BUB1 mitotic checkpoint serine/threonine kinase	− 3.071	− 2.581	− 3.272	− 4.774	− 5.280
BUB1B	BUB1 mitotic checkpoint serine/threonine kinase B	− 1.936	− 2.684	− 3.349	− 3.252	− 3.566

### Database validation of hub genes

**Figure 5:**
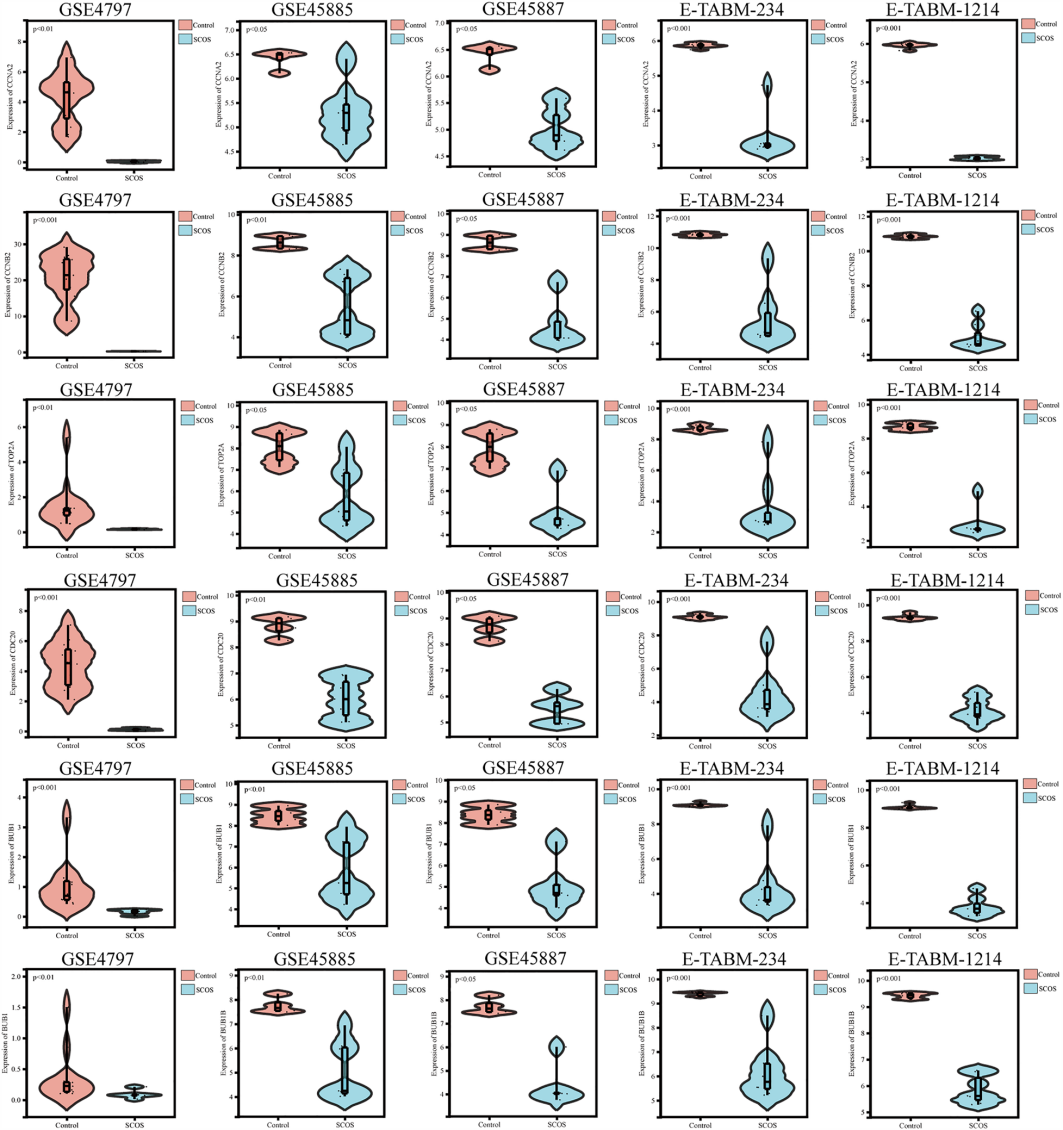
Validation of hub genes in microarray datasets. The expression of hub genes including CCNA2, CCNB2, TOP2A, CDC20, BUB1 and BUB1B was detected in GSE4797, GSE45885, GSE45887, E-TABM-234 and E-TABM-1214.

The consistent expression of the six hub genes was validated in the aforementioned datasets (GSE4797, GSE45885, GSE45887, E-TABM-234, E-TABM-1214). The violin plots demonstrated that CCNA2, CCNB2, TOP2A, CDC20, BUB1, and BUB1B were significantly decreased in the patients with SCOS compared to control patients (Fig. 5).

The data set GSE149512 was employed to validate the expression of the hub genes in SCOS. GSE149512 comprises 17 single-cell gene-expression profiles, including three patients with SCOS (LZ017, LZ018, LZ019) and four healthy individuals (LZ003, LZ013, LZ014, LZ015). A total of 17,157 testicular cells were obtained from normal adults and 21,218 testicular cells were obtained from patients with SCOS. The top 2000 highly variable genes were identified, and then the data set was normalized, the PCA dimensionality reduction analysis was conducted.

**Figure 6:**
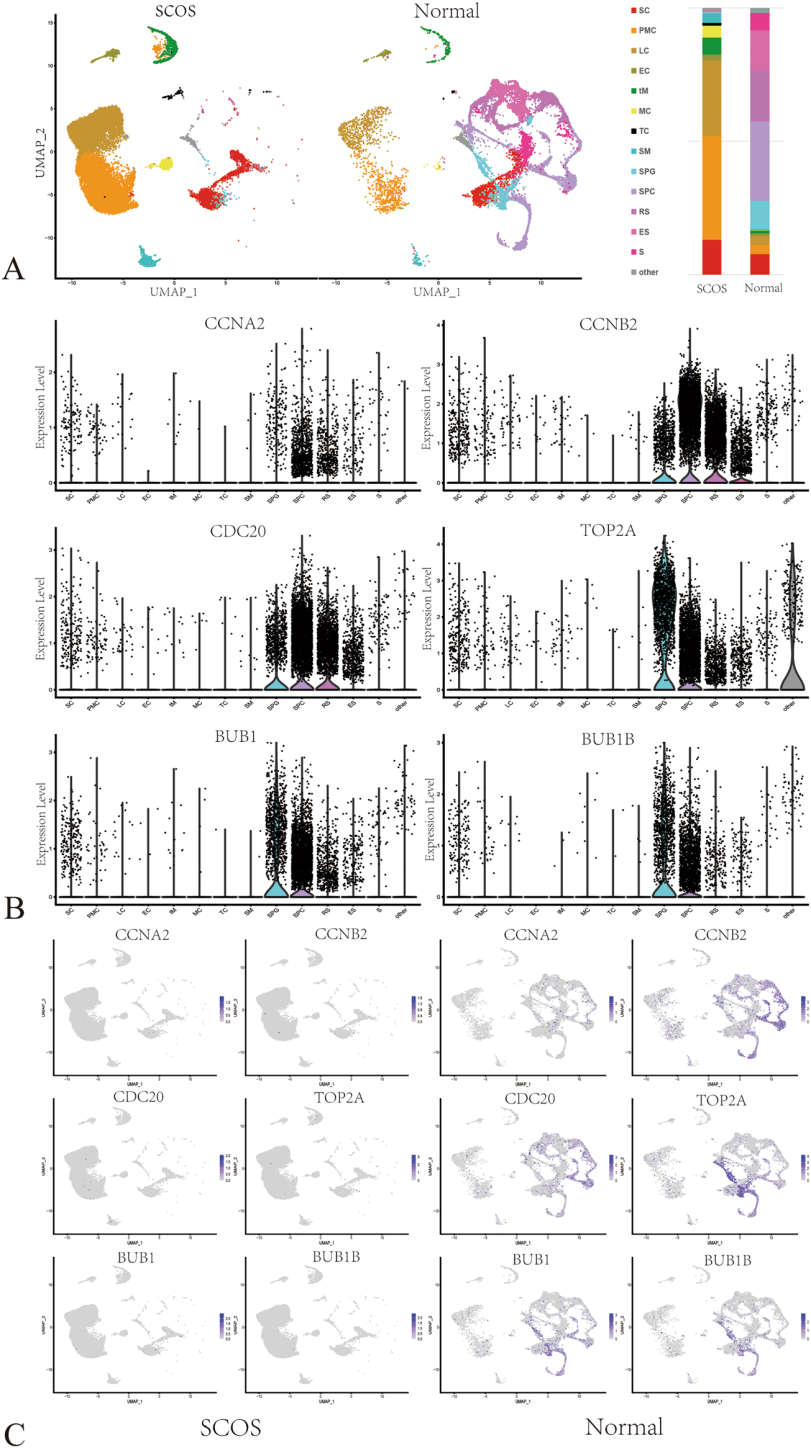
Validation of hub genes in Single-cell transcriptome dataset. (**A**) UMAP projections of scRNA-Seq data of SCOS sample (n = 3, 21,218 cells) and 4 normal adults (n = 4, 17,157 cells) split by cell type. The proportion of cell types in testes samples of SCOS and normal adults was shown in right part. Different cell types were shown with different colors as noted in the panel. (**B**) Violin plot of hub genes showing expression level in normal adults. (**C**) Distribution of the hub gene in different cell types of SCOS vs. normal adults in UMAP projections.

Using seurat for cell classification, 30 principal components were obtained, and the differentially expressed genes (logfc. threshold = 0.25) for each cluster were output as potential marker genes, as shown in the Supplementary Table 7. Using known markers, 14 cell clusters were identified that corresponded to germ cells and somatic cells in the testis, including Sertoli cells (SC), peritubular myoid cells (PMC), Leydig cells (LC), endothelial cells (EC), testicular macrophages (tM), mast cells (MC), T cells (TC), smooth muscle cells (SM), spermatogonial stem cells (SSC), spermatogonia (SPG), spermatocytes (SPC), round spermatids (RS), elongated spermatids (ES), and sperm (Fig. 6A). Few germ cells were identified in the testicular cells of SCOS, while the proportion of somatic cells (SC, LC, and PMC) was considerably increased. Violin plots and dot plots were constructed to display the expression patterns of the hub genes CCNA2, CCNB2, TOP2A, CDC20, BUB1, and BUB1B from normal testes were also shown in Fig. 6B and Supplementary Figure 1. These six hub genes were found to be primarily expressed in germ cells (SPG, SPC, RS, ES). Figure 6C shows the distribution of the hub genes in the different cell types of SCOS vs. normal adults in the UMAP projections.

### Clinical validation of hub genes

**Figure 7:**
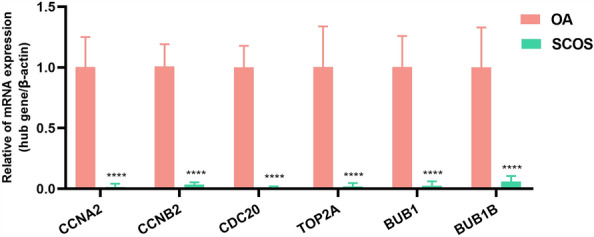
Clinical significance of Hub genes expression in patients with SCOS. Comparison of mRNA expression levels of six Hub genes (CCNA2, CCNB2, TOP2A, CDC20, BUB1 and BUB1B) in testicular tissues between patients with SCOS and OA. ****P < 0.0001.

Subsequently, the expression levels of the six hub genes were verified by RT-qPCR (Fig. 7). The results revealed that the mRNA expression levels of CCNA2, CCNB2, TOP2A, CDC20, BUB1 and BUB1B in the testicular tissue of patients with SCOS were significantly lower compared with those in the OA (****P < 0.0001).

### Construction of miRNA–mRNA networks and prediction of potential miRNAs

**Figure 8:**
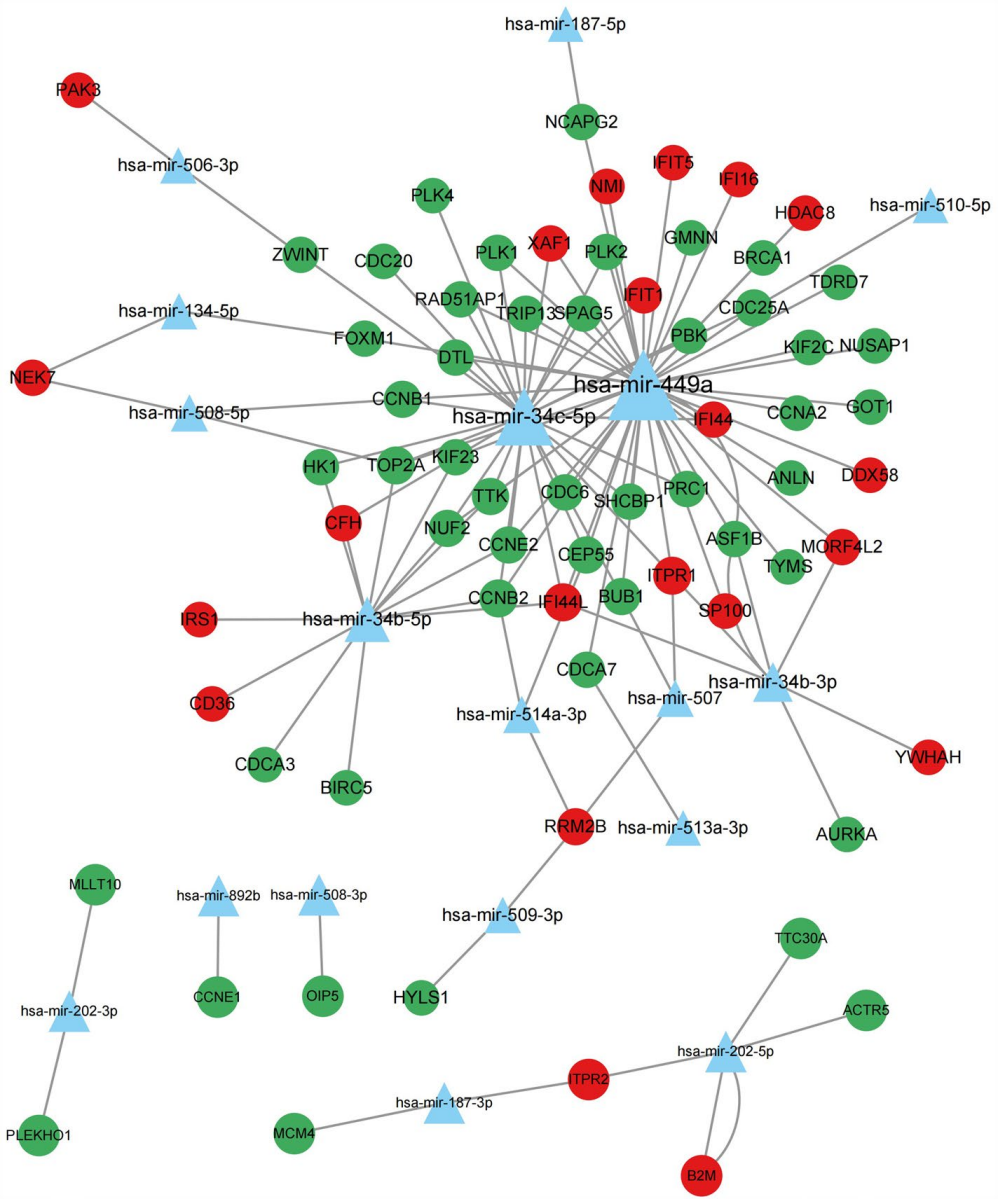
Integrated miRNA-mRNA interaction networks for the hub genes. Green and red circles represent down-regulated and up-regulated hub genes. Blue triangles represent miRNA which has connectivity with hub genes. The value of the degree is represented by the size of the node.

The miRNet database was used to predict the potential miRNAs of the hub genes. The 211 mRNAs of the hub genes obtained from the aforementioned PPI network were uploaded and 16 targeted miRNAs were found. Based on the prediction results, the miRNA and mRNA co-expression regulatory network was constructed by Cytoscape, which consisted of 87 nodes and 124 edges (Fig. 8). Combined with the enrichment functional analysis results of miRNA, it can be concluded that the germ cell proliferation pathway may be regulated by hsa-mir-449a, hsa-mir-34b, and hsa-mir-34c. In addition, four of the six hub genes were regulated by hsa-miRNA-449a and hsa-miRNA-34c-5p.

## Discussion

The histopathological types of NOA are associated with different patterns of gene expression, the related genes being involved in determining distinct molecular classes and metabolic pathways^28^_._ Therefore, directly comparing the gene expression profiles of NOA and control groups is inappropriate. Moreover, it is inappropriate to identified hub genes in SCOS using NOA related datasets without pathological classification. In this study, SCOS and control samples were screened from databases which included testicular histopathological examination by Johnsen Score. Currently, some researchers have reported that the hub genes such as CCNA2, TOP2A, and other genes are closely related to SCOS^29,30^, but the results are inconsistent for the different sources and platforms of datasets.

A series of bioinformatic analyses were performed on five independent gene chip databases of SCOS, and 601 shared DEGs between SCOS and normal spermatogenesis were obtained. Interestingly, the result of GO and KEGG pathways enrichment analysis indicated that the down-regulated DEGs were primarily enriched in sexual reproduction, reproductive process, male gamete generation, gamete generation, and multicellular organism reproduction. Meanwhile, the up-regulated ones were primarily enriched in immune response, immune system processes, and immune effector processes. Several studies have demonstrated that the system governing spermatogenesis includes immune cell types and testicular cells that affect each other and are to some extent mutually controlled^31^. Therefore, two hypotheses have been proposed regarding the cause of SCOS: (1) External factors induced to a severe immune reaction in the testicular microenvironment, resulting in the apoptosis of the germ cells; (2) Abnormal testicular somatic cells secrete immunoregulatory factors and thus, actively modulate the testicular immune response^32^. Our study reiterates the association between SCOS and immune response; however, the specific causality remains unestablished, necessitating further investigation for clarification.

Subsequently, using the MCODE and cytoHubba plug-ins of Cytoscape, six hub genes, including CCNA2, CCNB2, TOP2A, CDC20, BUB1, and BUB1B were eventually obtained. CCNA2 (Cyclin A2) controls both the G1/S and the G2/M transition phases of the cell cycle by forming specific serine/threonine protein kinase holoenzyme complexes with the cyclin-dependent protein kinases. Cyclin A2 is expressed in spermatogonia and pre-leptotene spermatocyte^33^. CCNB2 (Cyclin B2) binds the transforming growth factor beta RII and thus cyclin B2/cdc2 may play a key role in transforming growth factor beta-mediated cell cycle control. Additionally, CCNB2 has been identified and expressed in human testicular germ cells^34^. The Cyclin mRNA transcript ratios were found to be significantly decreased in patients with spermatogenic disorders^35^. TOP2A (DNA Topoisomerase II Alpha) encodes a DNA topoisomerase that controls and alters the topologic states of DNA during transcription, and plays a critical role in several important processes including chromosome condensation and chromatid separation. TOP2A and small ubiquitin-like modifier proteins were reported to regulate the dynamics of meiotic chromosomes in germ cells^36^. CDC20 (Cell Division Cycle 20) encodes as a regulatory protein in the cell cycle. It is required for two microtubule-dependent processes, nuclear movement before anaphase and chromosome separation. The missense mutation of CDC20 was found to be correlated with the pathogenesis of NOA in men^37^. BUB1 (BUB1 Mitotic Checkpoint Serine/Threonine Kinase) encodes a serine/threonine-protein kinase that plays a central role in mitosis. The encoded protein functions as the mitotic checkpoint complex and activates the spindle checkpoint. BUB1B (BUB1 Mitotic Checkpoint Serine/Threonine Kinase B) also encodes a kinase involved in the spindle checkpoint function. Both of them play the role in inhibiting the activation of the anaphase-promoting complex/cyclosome (APC/C) and are highly expressed in testicular germ cells in humans. BUB1 and BUB1B inhibit immune responses by promoting the secretion level of checkpoint molecules of the immune system^38^. The hub genes we obtained are all cell cycle-related genes. The expression of these genes is predominantly observed in male germ cells and is crucial for the process of spermatogenesis. Meanwhile, the low expression of cell cycle-related genes may induce the over-expression of immune response-related genes, which also confirms the results of previous pathway enrichment analysis.

To our knowledge, scRNA-seq allows for the analysis of extremely diverse cell populations in the testes at a single-cell resolution. To investigate the expression of the aforementioned six SCOS hub genes, their distribution of these genes across various cells in testicular tissue of SCOS vs. normal adults was analyzed. In the current study, the SCOS hub genes were validated through scRNA-Seq analysis. An efficient scRNA-Seq library construction method was applied to the SCOS and normal testes samples from dataset, which contained 21,218 and 17,157 cells, respectively. While these genes are also expressed in somatic cells, their predominant expression is observed in germ cells. The findings in scRNA-seq dataset is consistent with the validation in clinical samples. To some extent, using tissues containing different tissue types for differential expression analysis can reflect the expression differences in different tissues. However, the variation in expression is just linked, not causative. Thus, an alternative hypothesis exists, suggesting that the low expression of these genes, primarily observed in the germline, is not the underlying etiology of SCOS, but rather a result of germ cell depletion in this particular form of infertility. Further research is necessary to validate and expand on these specific causality.

MiRNAs are small non-coding RNAs that regulate gene expression by inhibiting mRNA translation or promoting mRNA degradation^39^. An integrated analysis of miRNAs and their target expression helps to reveal the regulatory pathways of miRNAs and identify functional miRNA-mRNA modules. In this study, a miRNA-target gene network was constructed, and the three most important miRNAs (hsa-mir-449a, hsa-mir-34b, and hsa-mir-34c) that interacted the most with the DEGs (46 DEGs, 20 DEGs, and 29 DEGs, respectively) were selected. The study of miRNA expression profiles in seminal plasma revealed that miR-34c-5p could be a potential noninvasive biomarker to diagnose patients with NOA and distinguish the different pathologic types of NOA^40^. Consistent with this conclusion, miR-34 and miR-449a families are the markers of low semen concentration and are crucial in spermatogenesis^41–43^. The miR-34 family, which is structurally similar to the members of the miR-449 family, has previously been shown to be highly expressed in and specific to germ cells^44^. The hub miRNA mediated and regulated diverse biological processes and signaling pathways (such as cell proliferation, cell apoptosis, actin filament network formation, and DNA damage repair) in SCOS. In the study of impaired spermatogenesis function, mRNA gene expression is upregulated owing to the down-regulation of miRNA gene expression^45,46^. Currently, testicular biopsy is an invasive technique utilized for diagnosing the spermatogenic capacity of patients, and a secure and noninvasive diagnostic approach is yet to be established. The miRNAs identified in this work may be integrated into the potential biomarkers for the rapid and noninvasive diagnosis of male infertility.

There were limitations of the present study. First, although the hub genes had been validated in the clinical testicular samples, further experimental validation of biomarkers in other tissues of SCO patients, particularly semen plasma, is needed for confirmation for noninvasive diagnosis of SCOS. Secondly, the hub genes with significant differential expression found in this study are all cell cycle-related genes highly expressed in Germ cell. However, the crucial role of somatic cells, particularly Sertoli cells, has not been focused on. A recent study revealed that maturation disorders in Sertoli cells are involved in SCOS, and inhibition of the Wnt signaling pathway promoted the maturation of these cells^7^. There have been enormous advanced in autophagy-related genes and confirms the importance of autophagy homeostasis in normal spermatogenesis^47^. Multiple factor disrupts spermatogenesis thoroughly, which in turn leads to the initiation and progression of SCOS.

## Conclusion

The current study identified and validated six novel hub genes (including CCNA2, CCNB2, TOP2A, CDC20, BUB1, and BUB1B) as potential biomarkers for SCOS. Further, a miRNA-gene network was constructed to predict the potential miRNAs (hsa-mir-449a, hsa-mir-34b, and hsa-mir-34c) involved in SCOS. These potential biomarkers that may be related to SCOS. However, it has not been proven that the differential expression of these biomarkers is the molecular pathogenesis mechanisms of SCOS. Our findings suggest that these biomarkers can be serve as clinical tool for diagnosis targets and may have some impact on the spermatogenesis of SCOS from a testicular germ cell perspective.

## Supplementary Information

Supplementary Table 1.

Supplementary Table 2.

Supplementary Table 3.

Supplementary Table 4.

Supplementary Table 5.

Supplementary Table 6.

Supplementary Table 7.

Supplementary Figure 1.

## Data Availability

The datasets supporting the conclusions of this article are available in the GEO (Gene Expression Omnibus) (https://www.ncbi.nlm.nih.gov/geo/) ArrayExpress and (https://www.ebi.ac.uk/biostudies/arrayexpress) repository. GSE4797: https://www.ncbi.nlm.nih.gov/geo/query/acc.cgi?acc=GSE4797, GSE45885: https://www.ncbi.nlm.nih.gov/geo/query/acc.cgi?acc=GSE45885, GSE45887: https://www.ncbi.nlm.nih.gov/geo/query/acc.cgi?acc=GSE45887, GSE149512: https://www.ncbi.nlm.nih.gov/geo/query/acc.cgi?acc=GSE149512. TABM-234: https://www.ebi.ac.uk/biostudies/arrayexpress/studies?query=E-TABM-234. E-TABM-1214: https://www.ebi.ac.uk/biostudies/arrayexpress/studies?query=E-TABM-1214. Further inquiries can be directed to the corresponding author.
