# Supplementary figures and images for: Identification and verification of potential biomarkers in sertoli cell-only syndrome via bioinformatics analysis
Source: Sci Rep. 2023 Jul 27;13:12164. doi: 10.1038/s41598-023-38947-4 (PMC10374527; doi:10.1038/s41598-023-38947-4)

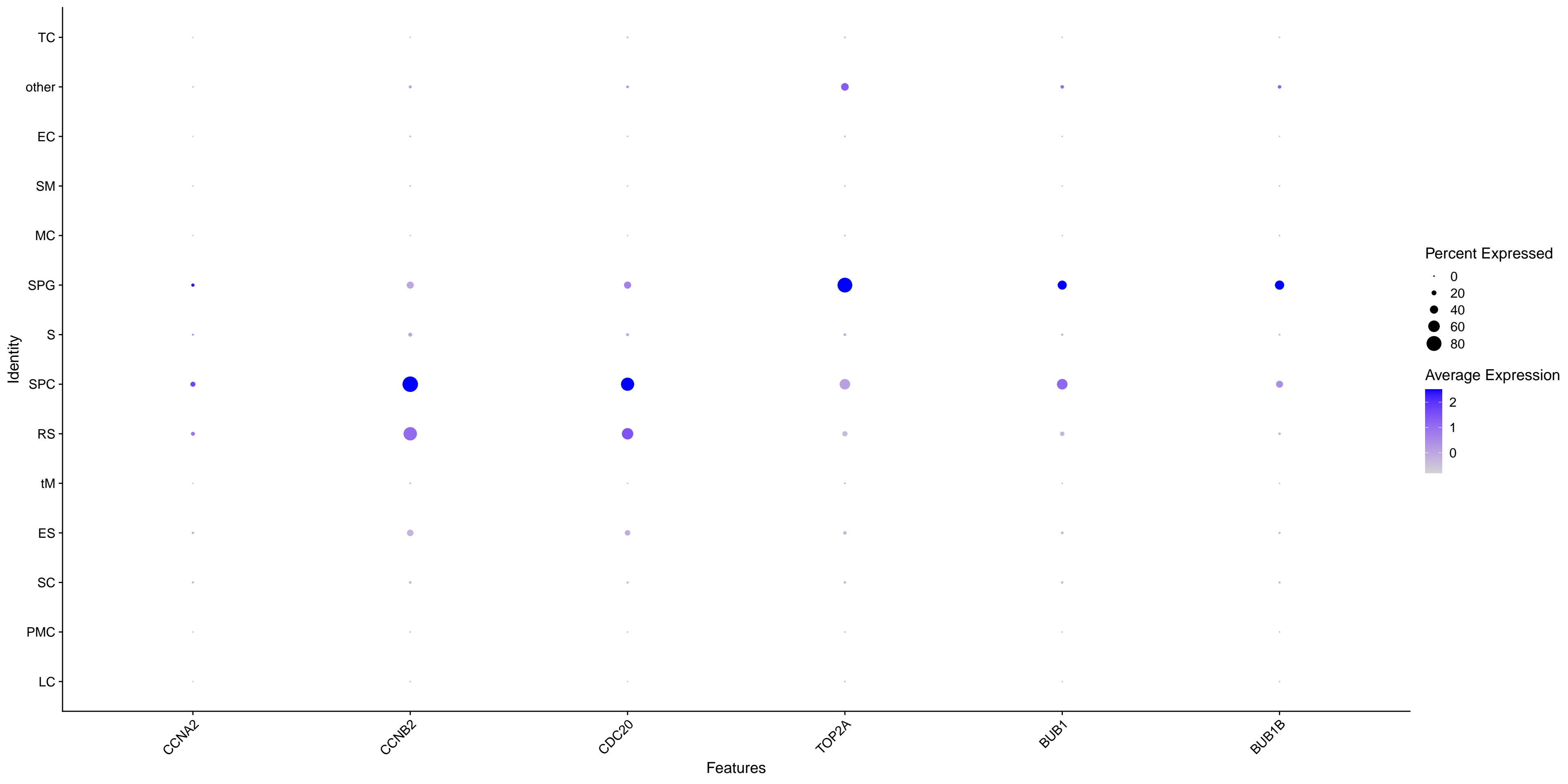

Supplement: Supplementary file 8 — Supplementary Figure 1. [file 41598_2023_38947_MOESM8_ESM.pdf]
